# Advances in gross tumor target volume determination in radiotherapy for patients with hepatocellular carcinoma

**DOI:** 10.3389/fonc.2024.1346407

**Published:** 2024-05-22

**Authors:** Kangning Meng, Guanzhong Gong, Rui Liu, Shanshan Du, Yong Yin

**Affiliations:** ^1^ Department of Graduate, Shandong First Medical University, Shandong Academy of Medical Sciences, Jinan, China; ^2^ Department of Radiation Physics, Shandong First Medical University Affiliated Cancer Hospital, Shandong Cancer Hospital and Institute (Shandong Cancer Hospital), Jinan, China

**Keywords:** hepatocellular carcinoma, radiotherapy, GTV determination, multimodal imaging, image segmentation - deep learning

## Abstract

Hepatocellular Carcinoma (HCC) is one of the most common malignant neoplasms. With the advancement of technology, the precision of radiotherapy (RT) for HCC has considerably increased, and it is an indispensable modality in the comprehensive management of HCC. Some RT techniques increase the radiation dose to HCC, which decreases the radiation dose delivered to the surrounding normal liver tissue. This approach significantly improves the efficacy of HCC treatment and reduces the incidence of Radiation-induced Liver Disease (RILD). Clear imaging and precise determination of the Gross Target Volume (GTV) are prerequisites of precise RT of HCC. The main hindrances in determining the HCC GTV include indistinct tumor boundaries on imaging and the impact on respiratory motion. The integration of multimodal imaging, four-dimensional imaging, and artificial intelligence (AI) techniques can help overcome challenges for HCC GTV. In this article, the advancements in medical imaging and precise determination for HCC GTV have been reviewed, providing a framework for the precise RT of HCC.

## 1 Introduction

Hepatocellular carcinoma (HCC) is one of the most prevalent malignant tumors (1, 2). In China, the incidence rate ranks fourth, and the mortality rate is second (3, 4). Consequently, the five-year survival rate is only 18% (5, 6). Treatment modalities for HCC typically include surgery, transarterial chemoembolization (TACE), radiotherapy (RT), liver transplantation, chemotherapy, biological targeted therapy, et al. (7).

As the accuracy of RT improved, the incidence of Radiation-induced Liver Disease (RILD) gradually decreased, leading to more frequent application of RT in HCC treatment (8, 9). RT can be used to treat all stages of HCC and serves as a crucial component of monotherapy or combined treatment approaches (10, 11). The initial critical step in RT involves precisely determining Gross Tumor Volume (GTV), which forms the foundation and prerequisite for assessing the efficacy of RT. Radiation oncologists earlier relied mostly on CT and MRI images for HCC GTV determination. With advancements in imaging techniques, new methods for HCC GTV determination have emerged. In this study, the progress in medical imaging and GTV determination for HCC RT has been reviewed.

## 2 The role of RT for HCC

Due to the unknown radiosensitivity of the liver and uncertainties about the occurrence of RILD, the application of RT in HCC treatment was limited (9). Currently, as RT accuracy improves, its status in HCC treatment continues to rise. In early 2018, the National Cancer Comprehensive Network (NCCN) revised its guidelines for unresectable HCC, changing the recommendation level for local RT from 2B to 2A (12). Due to the development of more precise RT techniques, such as Stereotactic Body Radiotherapy (SBRT), the risk of RILD has decreased, and the safety of External-beam Radiation Therapy (EBRT) for HCC has gained widespread recognition (9).

EBRT plays a key role, whether used independently or along with other treatment modalities (11). Kubo et al. (13) showed that the three-year and five-year Local Control (LC) rates for SBRT of small liver cancer remained at 100%. Huo et al. (14), summarized 11 randomized controlled trial groups comprising 2,577 HCC patients and found that the one-year survival rate was higher for those who underwent TACE combined with conventional fractionated RT compared to TACE alone. Similarly, Wei et al. (15) reported that the 24-month overall survival rate for HCC patients with portal vein tumor thrombus treated with EBRT in combination with surgical resection is higher than the survival rate after surgery alone. Some advanced HCC patients respond favorably to Immune Checkpoint Inhibitor (ICI) therapy, and the synergy between ICI and RT can amplify the immune response, improving Systemic Therapy Augmented by Radiotherapy (STAR) (16).

Due to its ability to overcome tumor location constraints, the application of Radiofrequency Ablation (RFA) is limited to early-stage HCC growing in key anatomical locations. EBRT can replace RFA or may be used along with RFA (11). Additionally, EBRT can reduce the size of tumors and stabilize patients awaiting liver transplantation, effectively serving as a “bridge” to liver transplantation (9).

## 3 Causes of HCC RT failure

The primary causes of RT failure in HCC are associated with issues concerning local control and the occurrence of RILD. Some studies have shown that both these factors are closely linked to inaccurate GTV determination.

Yoon et al. (17) found that tumor size was the only significant determinant of LC. Specifically, the three-year LC rate for patients with HCCs more than 3 cm was 14.7% lower compared to the rate for patients with lesions measuring 2 cm or smaller. This discrepancy occurred mainly because of the variance in the median total dose and GTV. The tolerance of the liver to radiation exhibits dose-volume effects, implying that a small portion of the liver tissue can withstand higher doses (18). As many HCC patients have a history of chronic liver disease, the available volume of normal liver function is limited. Consequently, as tumor size increases, adjustments must be made to GTV and the dose during RT to preserve normal liver function. This adjustment often results in a lower dose of GTV or a reduction in the volume of GTV, thus leading to suboptimal LC in patients with larger-volume HCCs (17).

RILD is one of the most severe complications of HCC RT, with a high mortality rate of 84%. It acts as a limiting factor in the application of RT in treating HCC, and no effective treatment is known (19, 20). To prevent RILD, the radiation dose administered to normal liver tissue needs to be controlled strictly. To achieve this, accurate determination of GTV is required. RILD was reduced following the adoption of SBRT (20, 21). In addition, Duan et al. (22) demonstrated that the use of multi-modal imaging techniques, such as Three-dimensional Computed Tomography (3D-CT), Four-dimensional Computed Tomography (4D-CT), and multi-parameter MR, for tumor determination resulted in significant differences in the determined tumor volumes. Specifically, compared to those derived from MR imaging, the median volumes of the Planning Target Volume (PTV) derived from 3D-CT and 4D-CT were 27.51% and 15.15% larger respectively. Consequently, there was a notable discrepancy in the median average doses of normal liver tissue: 15.97Gy, 12.89Gy, and 11.97Gy, respectively. These findings underscore the importance of accurate GTV determination, as it allows for a more precise determination of the expansion boundary for HCC, thereby reducing the radiation dose delivered to normal liver tissue and minimizing the risk of RILD. The precision of HCC GTV determination strongly influences the treatment effectiveness and the occurrence of RILD.

## 4 Challenges in determination the GTV of HCC

### 4.1 Display of HCC boundaries

Nearly all clinical guidelines recommend imaging-based diagnosis of HCC (23). The most valuable diagnostic modalities for HCC include multiphase-enhanced Computed Tomography (CT) or Magnetic Resonance Imaging (MRI) scanning. The American Association for the Study of Liver Diseases (AASLD) recommended combining arterial phase, portal venous phase, and delayed phase CT or MRI scans for diagnosing HCC and assessing treatment efficacy. The key characteristics of HCC lesions include “fast-in, fast-out”, delayed capsule enhancement, and high threshold values (24).

Irrespective of the RT method used, precise determination of GTV is an essential prerequisite for successful treatment. Separating the border of the tumor from the adjacent normal tissue is crucial for accurate GTV determination (25). The development of a Fibrous Capsule (FC) is a distinctive pathological characteristic of HCC, which occurs due to the interaction between the tumor and the liver. It generally serves as the boundary between HCC and the surrounding normal liver tissue. Its presence indicates a less aggressive nature of the lesion and serves as a crucial prognostic factor (26). Imaging examinations can clearly visualize the distinctive features of HCC lesions and provide greater insights into the relationship between the lesion and the surrounding normal liver tissue. However, the imaging and border representation of the lesion can vary significantly depending on the imaging examination method used (see **Table 1**). In cases of unencapsulated HCC, determining the boundary becomes more challenging.

**Table 1:** Advantages and limitations of different imaging methods for detecting HCC boundaries.

Imaging methods		Advantages	Limitations
CT	Dynamic CT Imaging	High diagnostic sensitivity and specificity (27).	Low soft tissue resolution and limited boundary display capabilities.
	DECT	Reveal differences in the HCC signal between reconstructed VMI and the iodine map, improving the HCC detection rate. Low keV levels (40 keV, 50 keV, and 70 keV) significantly improve HCC depiction and diagnostic capabilities (28).	
	ssDECT	Fusing single-energy spectrum images with optimal CNR and minimal noise can be used to attain arterial phase images that can clearly show liver tumors and anatomical structures, thus helping identify more and smaller HCCs (29).	
	pCT	Provide quantitative insights into arterial perfusion and assess the hemodynamic variations during the early stages of HCC (30). Play a qualitative role in HCC diagnosis predominantly (27).	
MR	DCE-MRI	High soft tissue resolution along with high sensitivity and specificity for HCC (31, 32). Tumor borders are clearly captured.	Low spatial resolution, long scanning time, and patient respiratory motion cause images to form artifacts easily.
	QDSA	Accurately measure arterial blood flow and improve the detection rate of HCC in the background of cirrhosis (33).	
	SWI	The positive rate of HCC border detection is 41.8% higher than MR conventional sequence (34).	
PET/CT		Displaying physiological and metabolic information, combined with traditional CT and MR, the diagnostic accuracy can be higher than 90% (35). The sensitivity of dual tracer combination is significantly higher than that of conventional CE-CT (93.8% vs 43.8%) (36).	Low sensitivity and limited diagnostic ability (37), low soft tissue resolution and limited boundary display ability (38).
PET/MR		Soft tissue has high contrast, provides physiological and metabolic information (39), and has high sensitivity and accuracy in diagnosing (40).	High cost and long acquisition time, not suitable for routine detection of HCC and RT simulation positioning.

DECT, Dual-energy CT; VMI, Virtual Monochromatic Images; ssDECT, single source Dual Energy CT; pCT, Perfusion CT; DCE-MRI, Dynamic Contrast-enhanced MRI; QDSA, Quantitative Digital Subtraction Angiography; SWI, Multi-breath-hold Two-dimensional Susceptibility-weighted Imaging; PET/CT, Positron Emission Tomography/Computed Tomography; CE-CT, Contrast-enhanced CT; PET/MR, Positron Emission Tomography and Magnetic Resonance.

### 4.2 Influence of respiratory movement

**Figure 1:**
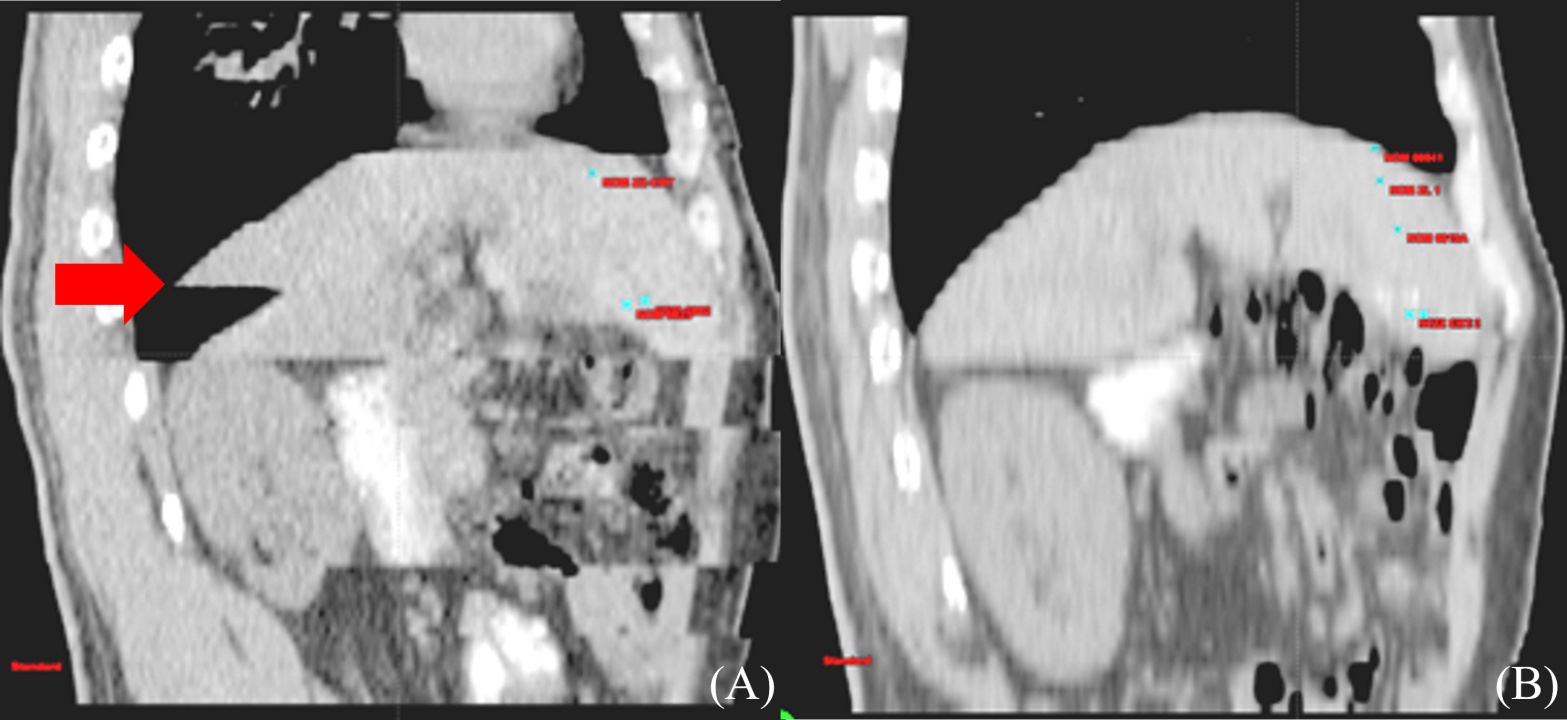
Differences of liver delineation imaging between free breathing and calm end-expiratory breath-hold CT scans. **(A)** CT sagittal imaging under free breathing **(B)** CT sagittal imaging at end-expiratory breath-hold phase.

Respiratory motion-induced position and shape variations of tumors strongly influence GTV determination. The liver moves when patients breathe due to its positional peculiarities, and the movement in the head and foot direction alone is 0.5–4.1 cm, which seriously affects the accuracy of HCC RT (41) (**Figure 1**). Balter et al. (42) recorded volumetric fluctuations of up to 12% between two distinct states: free breathing and breath-holding. Shimizu et al. (43), used High-speed Magnetic Resonance Imaging and found that when determining the PTV with a 10 mm safety margin, 19% of the PTV during expiration failed to include the Clinical Target Volume (CTV), while during inspiration, this figure increased to 36%. Respiratory motion showed a strong effect on HCC, introducing uncertainties in precise GTV determination. It also strongly contributed to local control failure in HCC.

## 5 Development of HCC GTV determination

The most widely used imaging modalities for the diagnosis and staging of HCC primarily include Ultrasonography (US), CT, and MRI. US is the predominant method for detecting and diagnosing HCC at an early stage and plays a key role in early HCC screening. However, for determining GTV of HCC, CT, MRI, PET/CT, and other imaging methods that can perform comprehensive whole-body contour imaging are used.

CT and MRI images of HCC patients are the most frequently used clinically for determining GTVs. Additionally, determining the biological target volume based on PET/CT images facilitates the comparison of variations in RT sensitivity between tumor and normal liver tissue, which can help in improving the effectiveness of RT. The consensus among experts is that multimodal imaging can significantly improve the accuracy of HCC GTV segmentation by supplementing anatomical and functional information.

### 5.1 HCC determination based on CT and MRI

**Figure 2:**
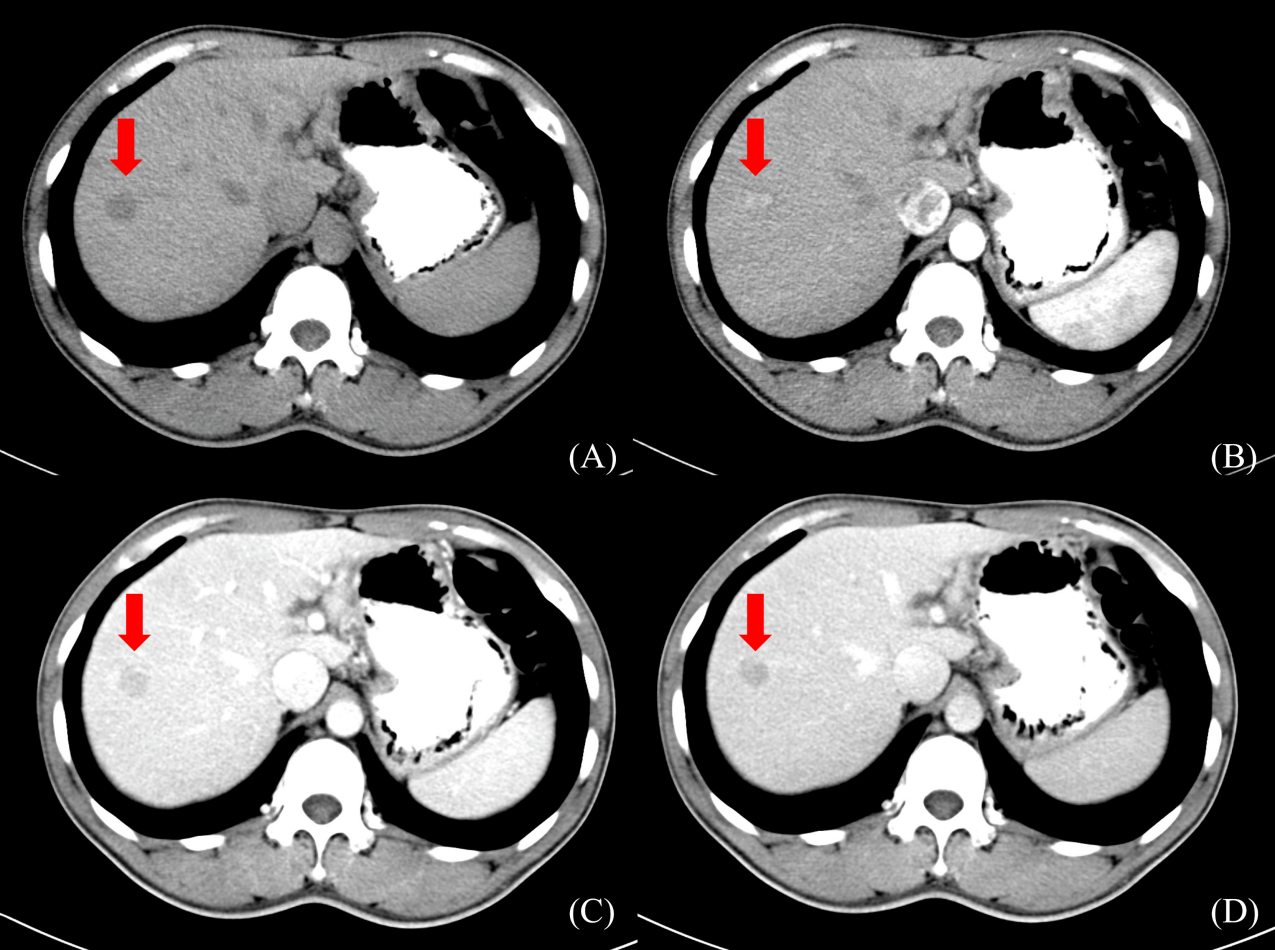
HCC CT border imaging. **(A)** CT scan without contrast **(B)** CT contrast scan in arterial phase **(C)** CT contrast scan in portal venous phase **(D)** CT contrast scan in delayed phase.

**Figure 3:**
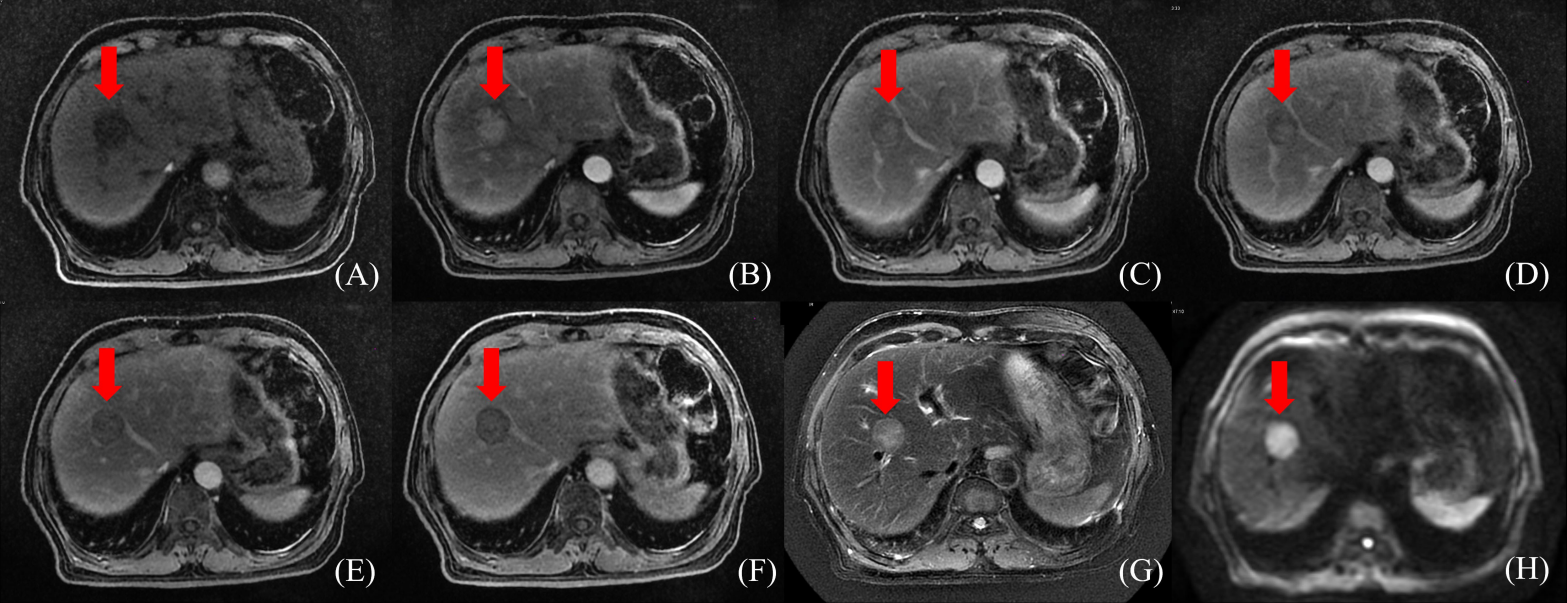
HCC MR border imaging. **(A)** T_1_-weighted Image without contrast **(B–F)** T_1_-weighted Image after contrast agent 15s,45s,75s,150s,10min **(G)** T_2_ –weighted Image with fat suppression **(H)** Diffusion Weighted Imaging (DWI).

**Figure 4:**
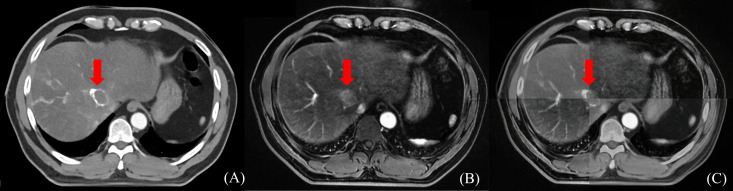
The boundary determination of HCC tumors using CT and MR registration. **(A)** CT contrast scan in arterial phase **(B)** MR T_1_-weighted arterial phase enhanced imaging **(C)** CT&MR rigid registration demonstration.

Radiation doses during HCC RT are calculated primarily based on CT images. However, the CT scans of many patients fail to distinctly determine tumor boundaries; this is a serious problem concerning GTV determination for HCC (44) (**Figure 2**). In contrast, MRI offers superior soft tissue resolution and provides greater anatomical details (45). It shows the tumor boundaries clearly and improves the precision of GTV determination (**Figure 3**). Integrating CT and MRI images for GTV determination can significantly improve the accuracy of determining the tumor boundary (**Figure 4**).

Ren et al. (46) used the mutual information method to show the significance of fusing CT and MRI images in determining HCC GTV. They stated that the GTV for HCC RT should be the sum of GTVs determination through both imaging modalities. Cheung et al. (25) introduced the Multisource Adaptive MRI Fusion (MAMF) method and showed that the fusion of only four MRI images (MRI T_1_WI venous phase, T_1_WI 19 min delayed phase, T_2_WI, and DWI) substantially improved the consistency of GTV determination, yielding an average Dice Similarity Coefficient (DSC) of 0.95 ± 0.02 among observers. Thus, MAMF is a promising approach for HCC RT GTV determination. Although the accuracy improves after CT and MRI images are fused, the technique does not address the issue of the effect of respiratory motion on GTV.

### 5.2 HCC determination based on 4D-CT and 4D-MRI

Four-dimensional imaging technologies, such as 4D-CT and Four-dimensional MRI (4D-MRI), can capture the respiratory motion curve of the patient and conduct scans based on their respiratory patterns to track the motion of GTV. Xi et al. (47) used 4D-CT to determine HCC GTVs and found a 19.4% reduction in the PTV compared to 3D-CT. This reduction protects more normal liver tissues and acts as a foundation for increasing the GTV dose.

However, 4D-CT imaging cannot effectively capture the timing of contrast agent injection; thus, enhanced 4D-CT scans cannot be obtained. Contrast-enhanced Three-dimensional CT (CE 3D-CT), which provides clear tumor boundaries for HCC, can complement the limitations of 4D-CT when the two approaches are combined. Xu et al. (48) performed deformable registration of these two types of images and investigated the relationship between the Internal Gross Tumor Volume (IGTV) post-registration and the average IGTV volume across 10 phases of 4D-CT. The results indicated that after registration, the GTV average in different 4D-CT phases increased by 36.29% (p < 0.05), and the IGTV volume increased by 19.91% (p < 0.05). This suggested that fusing arterial phase-enhanced 3D-CT with non-enhanced 4D-CT images can improve IGTV determination accuracy and prevent treatment failure due to insufficient GTV coverage.

Compared to 4D-CT, 4D-MRI provides better resolution of soft tissue images and can more accurately assess the range of motion of GTV. It is an ideal method for determining HCC GTVs. Chen et al. (49) compared GTV determination between 4D-CT and 4D-MRI. They found that compared to 4D-CT, 4D-MRI showed 25.04 cm^3^ and 22.44 cm^3^ lower values for GTV and Internal Target Volume (ITV), respectively (p < 0.01); also, 4D-MRI exhibited better repeatability. However, the clinical application of 4D-MRI for guiding HCC radiation therapy is still in its early stages.

### 5.3 HCC determination based on PET/CT

**Figure 5:**
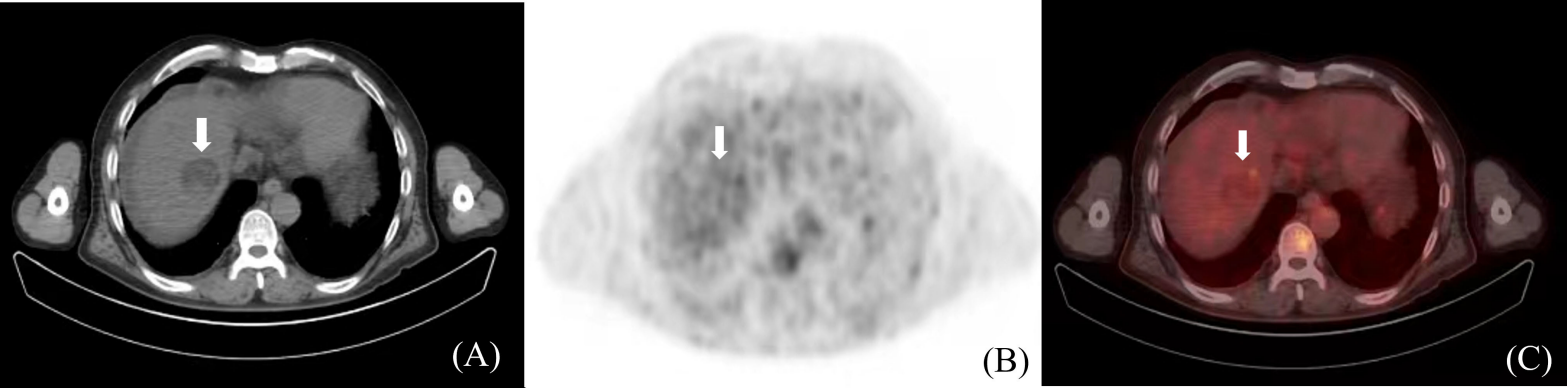
HCC PET/CT border imaging. **(A)** CT scan without contrast **(B)** PET images **(C)** PET/CT images.

Positron Emission Tomography/Computed Tomography (PET/CT) is a metabolic function imaging modality that can be used for the staging, diagnosis, and prognosis evaluation of malignancies. It dynamically displays HCC metabolism and reflects its biological activity. However, due to the low soft-tissue resolution of PET/CT, distinguishing tumor boundaries based only on PET/CT images is challenging. Studies on HCC tumor determination using PET/CT are limited (**Figure 5**). In the future, the design and application of HCC-specific tracers may improve the visualization of HCC tumor boundaries, thus offering a clinical basis for PET/CT in HCC GTV determination (50).

### 5.4 HCC determination based on PET/MR

Positron Emission Tomography and Magnetic Resonance (PET/MR) is an emerging technique characterized by the superior soft tissue contrast. It can also provide physiological and metabolic information through PET (39). PET/CT does not utilize respiratory gating technology during image acquisition, whereas PET/MR employs respiratory gating or breath-holding techniques during the MR sequence scan. Compared with PET/CT, PET/MR can enhance image quality by reducing respiratory motion artifacts and minimizing the rate of missing small lesions (39).

Parsai et al. (40) performed a retrospective analysis and found that PET/MR achieved a 100% lesion detection rate for characterizing malignant tumors, surpassing MRI or FDG PET/CT scans alone. This method offered the best performance, with sensitivity and accuracy of 91.9% and 94.7%, respectively.

However, despite its ability to offer metabolic information on HCC, PET/MR is not conducive to routine HCC detection or RT simulation positioning due to its limitations, which include high cost, prolonged acquisition time, and low FDG activity in well-differentiated HCC.

## 6 Respiratory motion solutions

To reduce the effect of respiratory motion on GTV determination, initially, a PTV was created with a generous external border on 3D-CT scans during free breathing. However, this approach had the risk of inconsistent external borders, extensive irradiation, and significant damage (51). With technological advancements, various motion management tools have emerged. Different clinical motion management methods are now available, including the Active Breathing Control (ABC) technique, Abdominal Compression (AC) technique, and respiratory gating technique (see **Table 2**). The AC technique is used extensively for controlling liver-lung direction tumor motion during SBRT (52). Additionally, 4D-CT or Cine-magnetic Resonance Imaging can assess any movement in the liver and compensate for the impact of respiratory motion (52).

**Table 2:** Summary of respiratory motion solutions.

Respiratory motion solutions	Study	Findings	Summary
ABC Technique	Eccles et al. (41)	It helps to locate the liver accurately and has good reproducibility of the liver position.	It can effectively reduce the influence of respiratory motion on GTV determination, it is highly demanding on patients, as it requires good respiratory function and cooperation.
	Zhao et al. (53)	It recorded a 32.26% reduction in PTV from 781 cm^3^ to 529 cm^3^ during free breathing.	
AC Technique	Herfarth et al. (54)	It can decrease tumor motion range by an average of 7 mm, effectively reducing the PTV external distance.	It can effectively reduce liver movement, but normal breathing rhythm and amplitude may change, increasing the movement along a specific axis of the tumor.
	Srisuthep et al. (55)	It can effectively reduce liver movement and decrease PTV by 33%.	
	Barton et al. (56)	It can reduce ITV by an average of 11.4%. However, 15.4% of patients experienced an abnormal increase in respiratory motion range and ITV.	
Gating Technique	Xi et al. (57)	It showed a significant reduction in the GTV of 54.4 ± 29.6 cm^3^ compared with the non-Gating group.	Although the Gating technique allows patients to breathe freely, it needs to establish a quantifiable relationship between the gating signal and tumor position that is repeatable. When patients have irregular breathing motion, the application of this technique may become more challenging and introduce greater uncertainty.
	Cheung et al. (58)	Using the Gating technique for GTV determination in HCC PET images reduced 21.48% of the GTV, on average, in the Gating group compared with the GTV of the non-Gating group.	

## 7 New techniques for determining HCC GTVs

Manually determining the GTV is time-consuming, laborious and has poor repeatability. The integration of artificial intelligence (AI) into GTV determination has increased due to advances in computer technology. It has not only improved the efficiency of physicians but also improved the consistency and accuracy of GTV determination, thus greatly improving the precision of RT. In the field of AI, image segmentation plays a prominent role in HCC GTV determination. Two commonly used methods in clinical practice are deep learning-based automatic segmentation and atlas-based segmentation.

### 7.1 Image segmentation based on deep learning

Image segmentation based on deep learning is gradually widely used in medical image processing, among which convolutional neural network (CNN) performs best in image segmentation tasks (59). Image segmentation methods based on deep learning are more stable and faster than manual determination. Doctors can make manual adjustments based on the segmentation results, and the deep learning model can self-train based on the doctor’s modification results, further improving segmentation speed and accuracy (60).

At present, due to the sufficient number of CT cases, low cost, and relatively convenient data collection, most automatic segmentation models for liver lesions are based on CT. The traditional CNN architecture used for liver tumor segmentation mainly includes full convolutional network (FCN), U-net and Segnet.

To optimize the CT segmentation results, Kushnure et al. (61) introduced a High-Level Feature Fusion and Recalibration UNet (HFRU-Net) based on the U-Net architecture for the automatic segmentation of HCC CT images. The method achieved a tumor segmentation DSC of 61.4%, which was comparable to the state-of-the-art methods.

To address the problem of poor end-to-end segmentation accuracy of FCN, Duan et al. (62) proposed a CT image liver tumor segmentation method based on convolutional multi-scale fusion FCN. The results in terms of volumetric overlap error (VOE), relative volume difference (RVD), average surface distance (ASD) and root mean square error (RMSD) were all significantly better than traditional FCN (18.36% vs 24.30%, 18.12% vs 18.24%, 7.64mm vs 14.64mm, 12.52mm vs 19.39mm). Compared with traditional FCN, it has high segmentation accuracy, good convergence and robust.

Compared with CT, Dynamic Contrast-enhanced MRI (DCE-MRI) has higher sensitivity for HCC diagnosis. Hence, Hänsch et al. (63) proposed a deep learning-based method using an anisotropic 3D U-Net and a multimodel training strategy for segmenting late-stage HCC tumors via DCE-MRI. Compared to the previous 2D U-Net, this approach increased DSC from 0.65 to 0.70. This study indicated that the model could improve the accuracy of automatic tumor segmentation for late-stage HCC patients. In addition, Zheng et al. (64) proposed a 4D deep learning model based on 3D convolution and convolutional long short-term memory (C-LSTM). When segmenting the 4D information of HCC DCE-MRI images, DSC and volume similarity are better than CNN_EF_ and CNN_LF_ models (0.825 vs 0.801, 0.797; 0.891 vs 0.849, 0.858, respectively).

**Figure 6:**
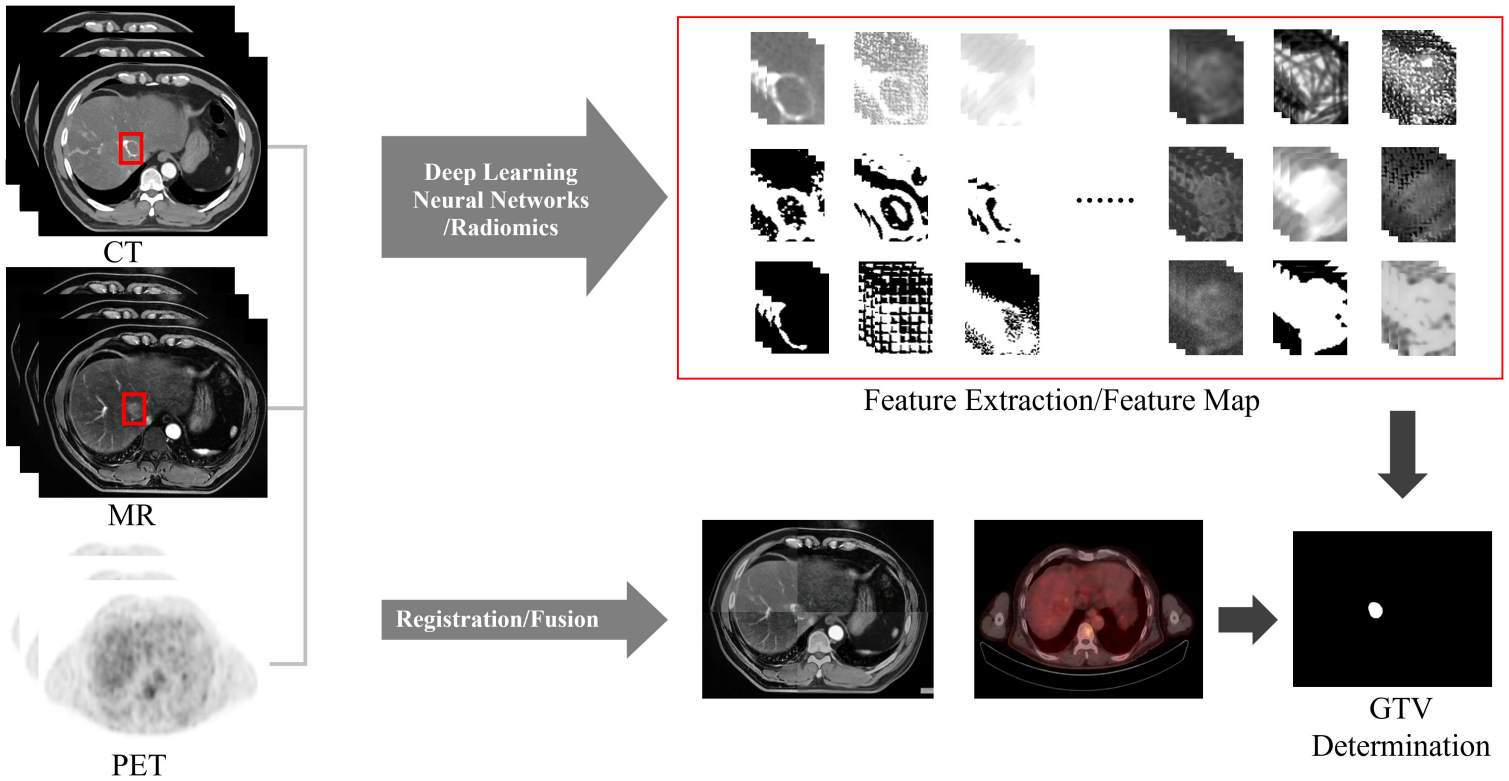
Workflow for determining GTV based on multimodal images and AI.

Due to the complex nature of HCC, blurred boundaries and low contrast caused by respiratory motion, a single imaging method cannot clearly display the tumor boundary, making automatic segmentation of liver tumors difficult. Therefore, when determining the GTV, the automatic segmentation method that combines multimodal images such as CT, MRI, and PET (**Figure 6**) will have greater advantages. And a unique training set can be established for each patient. Based on the patient’s image guidance information before each fraction of treatment, continuous self-training is provided to provide personalized segmentation methods for different patients. Multimodal and personalized segmentation methods will be one of the main development directions in the future.

### 7.2 Atlas-based image segmentation

Building an atlas library is a crucial step in atlas-based GTV determination. The process involves the creation of a database through the training of pre-contoured Organs At Risk (OARs) and GTVs. This database can then be used in clinical practice. Several Atlas-based Auto-segmentation (ABAS) software programs have been used in clinical RT. These programs have been extensively used in liver cancer and head and neck cancer RT.

Li et al. (65) developed a narrow-shell strategy, which uses liver contour point coordinates and contour adjacent image feature information for segmentation. It showed high accuracy in liver segmentation based on CT, 4D-CT, and CBCT images. The automatic determination of liver contours by Jaccard similarity was better by 90% to 96% compared to the manual determination made by doctors. This technique provides a practical solution for clinical automatic segmentation. Accurate segmentation of HCC tumors still faces significant challenges, one reason being the lack of standardized datasets (66). Quinton et al. (67) published the first dataset of DCE-MRI in 2023, which is expected to facilitate the development of atlas-based automatic segmentation models for HCC GTVs.

## 8 Conclusion and prospective

The incidence and mortality rates of HCC are increasing. Standardized and systematic treatment approaches are required to improve treatment efficacy while minimizing harm to patients. RT is one of the most promising treatment modalities and plays a vital role in precision treatment for HCC. Accurate determination of the GTV based on imaging is necessary for successful HCC RT.

In order to solve the shortcomings of manual determination, accurate tumor segmentation methods are the focus of current research. New and accurate tumor segmentation methods will help improve the accuracy and efficacy of RT and predict patient survival. Based on multisequence MR, CT, PET/CT images and 4D Imaging technology, the AI automatic segmentation model is expected to further improve the accuracy of HCC GTV identification and determination. Multimodal MR images contain rich tumor information, and AI and radiomics can deeply dig this information. This will be the development direction of automatic HCC segmentation in the future.

## Author contributions

KM: Writing – original draft. GG: Writing – review & editing. RL: Data curation, Writing – original draft. SD: Data curation, Writing – original draft. YY: Writing – review & editing.
